# Video assisted retroperitoneal debridement for infected pancreatic necrosis: A two port approach for difficult access

**DOI:** 10.1016/j.ijscr.2024.109740

**Published:** 2024-05-08

**Authors:** Daniel Gómez-Carrillo, Carlos Eduardo Rey Chaves, María Valentina Romero, Jaime Cruz, Roosevelt Fajardo, Francisco Díaz

**Affiliations:** a School of Medicine, Universidad de los Andes, Bogotá, Colombia; b Estudiante de posgrado Cirugía General, Pontificia Universidad Javeriana, Facultad de Medicina, Bogotá, Colombia; c Pontificia Universidad Javeriana, Bogotá, Colombia; d Department of Surgery, Hospital Universitario Fundación Santa Fe de Bogotá, Colombia

**Keywords:** Acute necrotizing pancreatitis, Infected necrotizing pancreatitis, Video-assisted retroperitoneal debridement, Step-up approach

## Abstract

**Introduction and importance:**

The incidence of acute pancreatitis varies globally, and its rates are increasing. Timely intervention in cases of infected necrosis is crucial to effective management. The landscape of acute pancreatitis management has undergone transformation through adopting a “step-up” strategy, accentuating the shift towards minimally invasive techniques.

**Case presentation:**

A 63-year-old patient with acute pancreatitis and infected pancreatic necrosis underwent a challenging yet successful treatment using video-assisted retroperitoneal debridement employing a two-port approach facilitated access for an intricate area. The procedure, performed 45 days after admission, effectively reduced peripancreatic collections, demonstrating the efficacy of this approach in managing complex cases of infected pancreatic necrosis.

**Clinical discussion:**

The management of acute pancreatitis has evolved towards a comprehensive strategy involving early hydration, nutritional support, effective pain management, and interventions. Infected pancreatic necrosis poses a serious complication, with minimally invasive techniques such as video-assisted retroperitoneal debridement (VARD) emerging as preferred options. The efficacy and safety of VARD in complex cases are highlighted, although challenges persist, especially in extensive necrosis.

**Conclusion:**

The VARD procedure, a key component of the step-up approach, exhibits a remarkable safety profile, substantially reducing postoperative complications and mortality compared to open surgical counterparts. However, challenges persist in managing cases of infected Walled-Off Necrosis with deep extension, necessitating carefully considering a minimal-access approach. We report our experience using the VARD in a two-port approach.

## Highlights

• Infected pancreatic necrosis poses high morbidity and mortality risks.
• Management evolution: Shift from traditional open surgery to "step-up" strategy using minimally invasive techniques.
• A 63-year-old patient with acute pancreatitis underwent video-assisted retroperitoneal debridement.
• Timing is key: Delay intervention in sterile necrosis. Inmmediate action needed for infected necrosis to reduce mortality.
• Managing infected necrosis is tough, sometimes needing extra open necrosectomy; consider minimal-access approaches.

## 1 Introduction and importance

The incidence of acute pancreatitis displays considerable variability across studies, driven by geographical and etiological factors. Research indicates an upward trend in incidence rates, surpassing 70 cases per 100,000 population annually in certain instances [1].

Understanding the clinical spectrum of acute pancreatitis is pivotal for effective management. While the majority experience mild attacks aligning with the Atlanta classification, approximately 20 % progress to severe episodes marked by necrosis of pancreatic or peripancreatic tissue, organ failure, and mortality [[1], [2], [3], [4], [5]]. Pancreatic necrosis not only contributes to increased morbidity and mortality but also escalates the risk of infection [3,4]. Infected pancreatic necrosis necessitates prompt and targeted interventions, often involving surgical or radiologic means for debridement and drainage [1,4]. Studies, such as the one conducted by Götzinger et al., underscore the significant contrast in outcomes based on the success of debridement. Failure to clear necrosis through surgery resulted in a 100 % mortality rate, while successful debridement significantly lowered the mortality rate to 19 % [[1], [2], [3], [4]].

The landscape of acute pancreatitis management has witnessed significant evolution over the years. Traditionally, open surgical debridement/necrosectomy was the primary intervention but was associated with increased composite endpoints of death or severe complications [4]. The techniques employed for necrosectomy in acute pancreatitis are varied. The contemporary approach favors a “step-up” strategy, sequentially employing minimally invasive techniques such as percutaneous catheter drainage, endoscopic transluminal debridement/necrosectomy, video-assisted retroperitoneal debridement, and sinus tract endoscopy [1,3,4]. Percutaneous or endoscopic drainage is the initial step, often followed by more targeted interventions such as endoscopic transluminal necrosectomy, sinus tract endoscopy for debridement, and video-assisted retroperitoneal debridement [[1], [2], [3], [4], [5], [6], [7]].

The comprehensive management of acute pancreatitis is undergoing a transformative evolution. Advances in understanding the varying incidence rates, the clinical spectrum, the role of pancreatic necrosis, mortality considerations, the evolution of management approaches, the timing of intervention, and diverse approaches to necrosectomy collectively contribute to a more nuanced and effective approach to this challenging medical condition.

Hence, we present the case of a 63-year-old patient who required a video-assisted retroperitoneal debridement of an acute necrotic collection and pancreatic necrosectomy via a hybrid video-assisted with a two-port approach technique.

## 2 Case presentation

After ethical and institutional approval, previous informed consent was filled following scare guidelines [8].

A 63-year-old patient with a past medical history of arterial hypertension presented to our institution's emergency department (ER) with a one-hour record of moderate to severe epigastric pain that began after eating. The patient exhibited tenderness in the epigastrium and right hypochondrium on physical examination. Consequently, the patient was admitted for symptomatic relief and evaluation of his abdominal pain, suspecting a hepatobiliary pathology. Upon admission, the following laboratory results were reported: complete blood count (CBC): white blood cells 10,500/mm3, neutrophils 41.9 %, lymphocytes 44 %, hemoglobin 16 g/dL, hematocrit 46.7 %, platelets 226,000/mm3; creatinine 0.84 mg/dL; aspartate aminotransferase (AST) 36 U/L; alanine aminotransferase (ALT) 59 U/L; total bilirubin 0.64 mg/dL; direct bilirubin 0.10 mg/dL; indirect bilirubin 0.54 mg/dL, and amylase 1638 U/L. Additionally, a hepatobiliary ultrasound was performed, revealing an enlarged liver with a longitudinal diameter of 178 mm and diffuse increase in echogenicity due to fat infiltration; a distended gallbladder with thin walls, no stones identified inside; negative Murphy's sign; the pancreas (body) had a typical ultrasound appearance, and the intra-extrahepatic bile duct was of normal caliber. Given the significantly elevated amylase levels, which were more than three times the laboratory reference range, the patient was diagnosed with acute pancreatitis, and a biliary origin was ruled out based on the laboratory results. Calcium and triglyceride levels were then assessed, resulting in values of 1.15 millimol/L and 172 mg/dL, respectively.

**Fig. 1:**
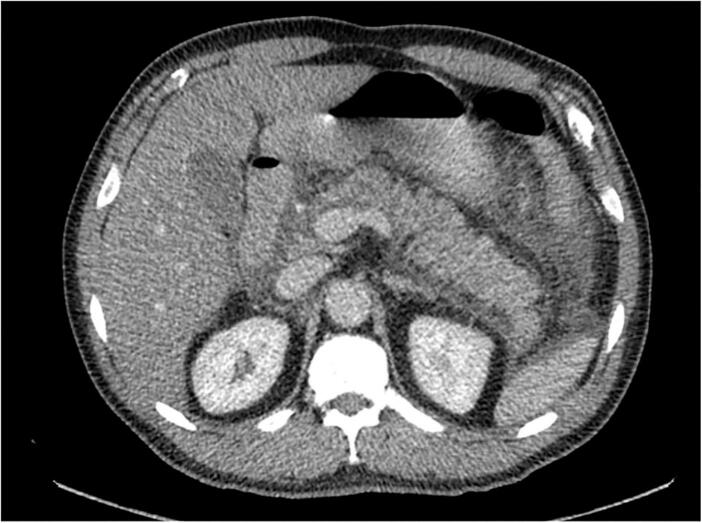
Enlarged pancreas, striation of the peripancreatic fat and peripancreatic free fluid, findings compatible with edematous pancreatitis.

Further evaluations were conducted to complete risk assessment scales, and an abdominal computed tomography (CT) scan was requested. The CT scan revealed signs suggestive of edematous pancreatitis with free peripancreatic fluid extending to the left paracolic gutter without the presence of collections or necrotic areas (Fig. 1). The patient was diagnosed with acute pancreatitis with an Apache score of 4 points and a Marshall score of 1. However, the etiology remained to be determined as it had been ruled out as being of biliary, alcoholic, hypercalcemic, or hypertriglyceridemic origin.

On the following day, the patient was transferred to the intensive care unit (ICU) due to tachycardia, oliguria, persistent systemic inflammatory response, and elevated lactate levels. High-flow supplemental oxygen was initiated due to a secondary restrictive pattern with a higher probability of left pleural effusion in the context of acute pancreatitis. Nasogastric tube placement and parenteral nutrition were also initiated.

Since the etiology of pancreatitis had not been established, a magnetic resonance cholangiopancreatography (MRCP) was requested one week after admission. The MRCP identified significant diffuse inflammatory changes in the pancreas, suggestive of edematous pancreatitis with poorly defined peripancreatic laminar collections and no pancreatic duct dilation. The biliopancreatic junction appeared normal; the gallbladder was distended with sludge but there were no signs of choledocholithiasis. The interventional radiology team assessed the findings and determined that drainage was not feasible at that time due to insufficiently formed collections.

The patient presents clinical deterioration, fever spikes, and elevated inflammatory markers; it was suspected that the patient's deterioration might be due to superinfection of acute pancreatic collections.

**Fig. 2:**
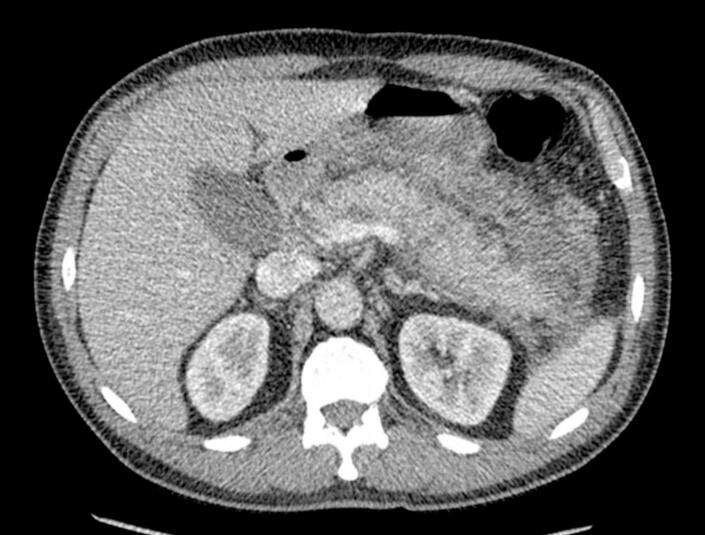
Unorganized, ill-defined peripancreatic collections adjacent to the tail (100 mm in greatest diameter), and multiloculated collection adjacent to the head (56 mm).

As a result, twelve days after admission, a new abdominal CT scan was requested. This scan revealed persistent signs of acute edematous pancreatitis with multiple poorly defined peripancreatic collections, with the largest adjacent to the pancreatic tail measuring 96 mm (Fig. 2). These findings were discussed with the interventional radiology team, who considered that drainage was not feasible then and recommended follow-up with MRCP, as the pancreatic collection was compressing the superior mesenteric vein. So, another MRCP was done. It showed inflammatory processes of the pancreas with poorly defined peripancreatic changes, likely hemorrhagic-necrotic in nature, predominantly anterior and inferior to the distal aspect of the pancreas and anterior and superior to the pancreatic head.

Consequently, the case was presented at the general surgery medical board meeting in collaboration with the interventional radiology team. It was concluded that the patient would not benefit from surgery. One month after the patient's admission, a catheter placement for percutaneous drainage of the collection was done. Following the drainage, the presence of infection in these collections by *Escherichia coli* with an IRT resistance pattern was confirmed, prompting targeted antibiotic therapy under the guidance of the infectious disease specialist.

However, considering the time course and the patient's stagnant condition despite the percutaneous drainage, following the stepwise approach to locally infected pancreatic complications, another medical board meeting was convened. During this meeting, a new evaluation of previously requested images was conducted. An extensive necrotic collection with signs of local infection in the second abdominal CT scan was identified, primarily between the neck and tail of the pancreas, with another area suggesting necrosis towards the pancreatic head that was not connected to the rest of the collection. Initially, minimally invasive approaches (video-assisted retroperitoneal and endoscopic) were considered, but the gastroenterology service determined that endoscopic drainage was not a viable option at that moment. Therefore, the proposal was to perform a video-assisted retroperitoneal approach (VARD).

**Fig. 3:**
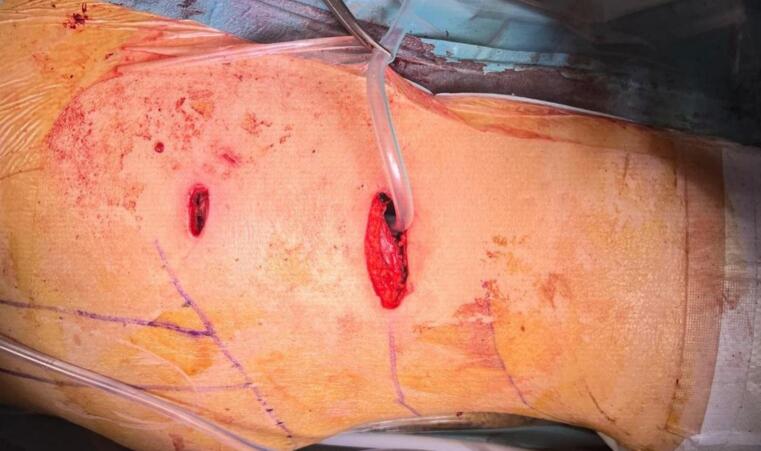
Surgical approach.

**Fig. 4:**
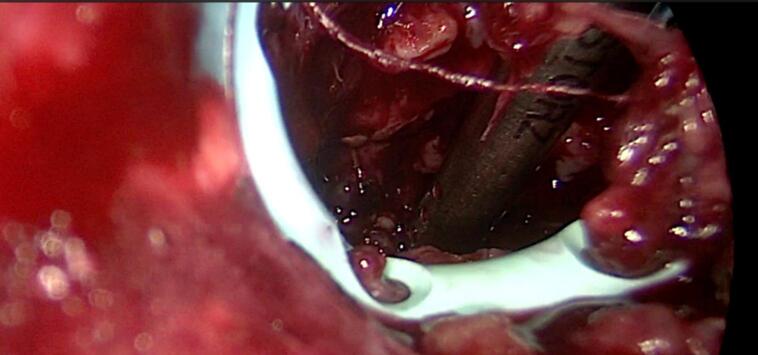
Pancreatic necrosectomy, debridement and lavage employing video-assisted techniques.

**Fig. 5:**
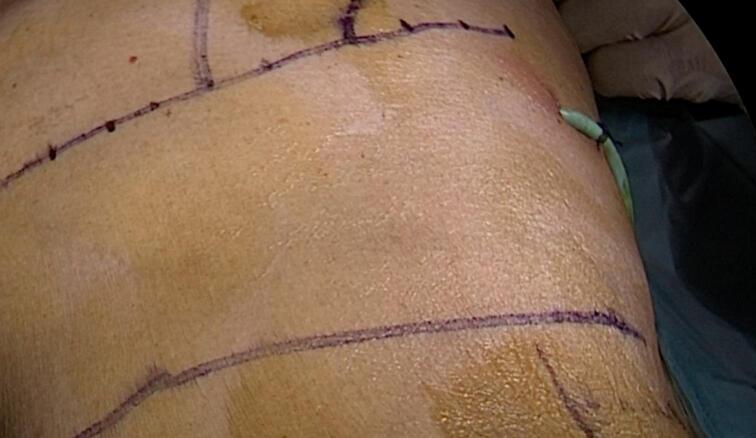
Drainage tube introduced.

Forty-five days after the patient's admission, a video-assisted retroperitoneal debridement of the acute necrotic collection, exploration of the retroperitoneal space via a hybrid video-assisted approach, and pancreatic necrosectomy via a hybrid video-assisted approach were performed. An incision was made at the left lumbar level below the costal margin on the midaxillary line, followed by dissection through the planes until identifying the transverse abdominal muscle (Fig. 3). The dissection continued until the retroperitoneal fat was identified, and digital dissection of the space was performed until the pigtail drain was palpated. The drainage pathway of the pigtail was accessed with blunt dissection. An optical trocar was introduced to visualize the pigtail drain and follow its trajectory to identify the cavity in the retroperitoneal space with hemorrhagic-purulent characteristics. Subsequently, the cavity was lavaged with 1500 cc of normal saline. Pancreatic necrosectomy and debridement were performed using video-assisted techniques until healthy tissue was visible (Fig. 4). The pigtail drain was then removed under laparoscopic vision, and a larger drainage tube (20 French chest tube) was introduced under direct vision, which remained in the previously described cavity (Fig. 5).

**Fig. 6:**
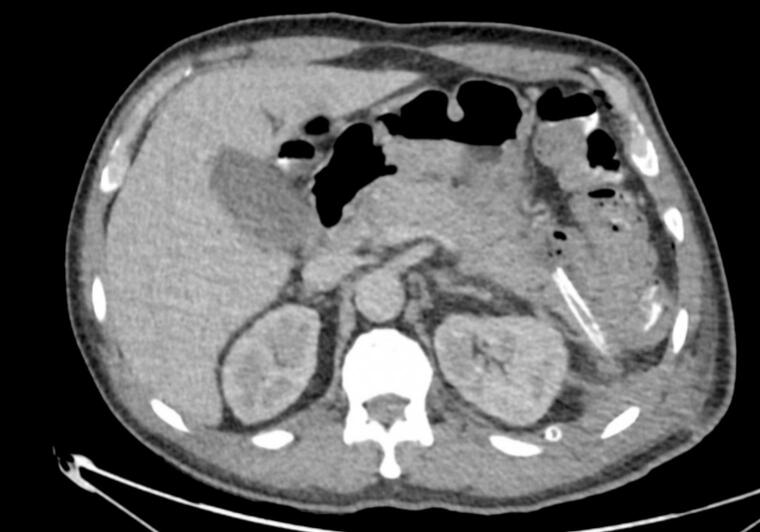
75 % resolution of peripancreatic collections, partial visualization of drainage catheter adjacent to the tail of the pancreas.

During the days following the procedure, there was a drainage of dark bloody fluid. Three days after the surgery, oral intake was initiated with adequate tolerance and a new abdominal CT scan was requested. It was noted that the peripancreatic collections in the neck of the pancreas with extension to the root of the mesentery and in the tail of the pancreas had decreased in size compared to the previous study. The drainage catheter was visualized in the collection adjacent to the tail of the pancreas (Fig. 6). Additionally, there was asymmetric thickening of the walls of the antral-pyloric region and the transverse colon, suggestive of inflammation due to the proximity of the peripancreatic and retroneumoperitoneal inflammatory changes, and gas bubbles in the extraperitoneal space, attributable to post-surgical changes.

Subsequently, due to adequate pain management and tolerance to oral nutrition, even with the drain, the patient was discharged 56 days after admission and on the 10th postoperative day. 20 days after discharge from the hospital, at the follow-up appointment, the drain was removed. After 4 weeks a laparoscopic cholecystectomy was performed without complications; the peritoneum was found to be healthy and unaltered. It is worth noting that, despite the patient's chronic cholecystitis with multiple stones and biliary sludge, the procedure was performed with excellent results and prompt patient recovery, allowing for a return to normal activities. No complications were evidenced at the follow-up appointments at 30, 60, and 90 days.

## 3 Clinical discussion

The landscape of acute pancreatitis management has undergone a nuanced evolution, grounded in four fundamental tenets: early intravenous hydration, optimal nutritional support, effective analgesia, and targeted interventions [9]. In our patient, a comprehensive treatment approach was adopted, encompassing fluid resuscitation, intravenous antibiotic therapy, nasal-jejunal enteral nutritional support, and meticulous pain control.

Nevertheless, as outlined by the Atlanta consensus (1992), there may be local applications associated with this clinical condition, such as encapsulate peripancreatic acute liquid collections, acute necrotic collection, pancreatic pseudocyst, walled-off necrosis, and infected necrosis [10]. A salient complication arising from pancreatic inflammation is infected pancreatic necrosis, historically associated with a mortality rate of up to 70 % despite surgical management [11].

Within the realm of necrotizing pancreatitis (NP), necrosis delineates into acute necrotic collections (ANCs) and walled-off necrosis (WON), each with distinctive clinical trajectories [3]. The latter has a lengthy clinical course, and the management may be even more challenging in the presence of an infection in the necrotic bed, as in this case. In such instances, it is highly advised to employ invasive measures to implement drainage, debridement, or necrosectomy on the necrotic collection [3,4].

However, treatment strategy has perceptibly shifted from conventional open surgical management to judiciously embraced minimally invasive techniques, encompassing both surgical and endoscopic modalities as stated in the step-up approach. This entails sequential interventions, including percutaneous catheter drainage, endoscopic transluminal debridement/necrosectomy, video-assisted retroperitoneal debridement, and sinus tract endoscopy. Minimally invasive drainage procedures are usually effective in resolving sepsis and early multi-organ failure in most patients with infected pancreatic necrosis [2,3,9,12]. However, since the patient did not recover satisfactorily despite this procedure, we advocate the performance of VARD.

The ascendancy of endoscopic techniques of drainage and debridement is fueled by the sobering complication and mortality rates attendant to surgical interventions. A discerning comparative study underscored the pronounced superiority of the minimally invasive step-up approach, markedly reducing major complications, mortality, long-term sequelae, and healthcare resource utilization in patients with necrotizing pancreatitis and secondary infection [3,4,11,12]. Particularly, the results of the VARD procedure evinced an impeccable safety profile, characterized by markedly diminished postoperative complications and mortality compared to their open surgical counterparts [3,4,11]. Accordingly, the step-up approach emerges as a linchpin in averting major abdominal surgery, mitigating associated complications, and forestalling protracted postoperative recovery.

A critical consideration in managing pancreatic necrosis is the timing of intervention. While there is consensus that delayed intervention is preferable, the urgency increases when the infection is apparent. Monitoring patients with significant pancreatic necrosis for signs of infection and intervening promptly is now common practice [3,6]. In the absence of significant symptoms, Sterile necrosis may not require routine debridement. However, infected necrosis, developing over time and peaking around 3 weeks after disease onset, demands immediate attention due to its association with increased mortality [1,3,4,7]. Hence, a deferral until the fourth week from symptom onset to curtail complications and mortality rates is recommended. In our scenario, the procedure was performed six weeks after admission.

However, in the intricate terrain of infected WON with deep extension (iWONde) or when the necrosis extends to the right of mesenteric vessels, minimal-access approaches grapple with formidable challenges, often necessitating iterative interventions or, in certain instances, resorting to supplementary open necrosectomy [3]. Therefore, open necrosectomy has come to be considered to provide complete debridement in a single procedure, which can be advantageous, especially in generalized necrosis [5]. The decision to opt for an anterior two-port approach instead of the classic description of VARD of the technical difficulty regarding the percutaneous drainage; for that reason and to have a secure entry to the retroperitoneum and into the necrotic tissue, a two-port approach was required, nevertheless the minimally invasive approach was maintained, and the technical characteristics remains the same, with safety and effectiveness.

## 4 Conclusion

The dynamic landscape of acute pancreatitis management has witnessed a notable evolution, with a paramount emphasis on early intervention, particularly in cases involving infected necrosis. Adopting a “step-up” strategy has underscored a significant paradigm shift, favoring the utilization of minimally invasive techniques, as exemplified in this case. The VARD procedure, a key component of the step-up approach, exhibits a remarkable safety profile, substantially reducing postoperative complications and mortality compared to open surgical counterparts. However, challenges persist in managing cases of infected Walled-Off Necrosis with deep extension, necessitating carefully considering minimal-access approaches and potential supplementary open necrosectomy.

## Provenance and peer review

Not commissioned, externally peer-reviewed.

## Consent

Written informed consent was obtained from the patient for publication of this case report and accompanying images. A copy of the written consent is available for review by the Editor-in-Chief of this journal by request.

## Ethical approval

Ethical approval of the institutional committee was obtained previous publication.

## Funding

This research did not receive any specific grant from funding agencies in the public, commercial, or not-for-profit sectors.

Research registration number

None.

## Guarantor

Daniel Gómez-Carrillo.

## CRediT authorship contribution statement

Daniel Gómez-Carrillo: Participated in drafting the article and revising it critically for important intellectual content. Made substantial contributions to conception and design, acquisition of data, analysis, and interpretation of data.
Carlos Eduardo Rey Chaves: Participated in drafting the article and revising it critically for important intellectual content. Made substantial contributions to conception and design, acquisition of data, analysis, and interpretation of data.
María Valentina Romero: Participated in drafting the article and revising it critically for important intellectual content.
Jaime Cruz: Participated in drafting the article and revising it critically for important intellectual content.
Roosevelt Fajardo: Participated in drafting the article and revising it critically for important intellectual content.
Francisco Díaz: Participated in drafting the article and revising it critically for important intellectual content.

All authors have revised and approved the final version of the manuscript.

## Declaration of competing interest

The authors do not declare any conflict of interest.
